# Frequency of Mismatch Repair Protein (MMRP) Deficiency among Young Jordanians Diagnosed with Colorectal Carcinoma (CRC)

**DOI:** 10.1155/2020/5632984

**Published:** 2020-04-23

**Authors:** Bayan Maraqa, Ghassan Al-Shbool, Osama Abu-Shawer, Mamoun Souleiman, Osama Alshakhatreh, Amal Al-Omari, Hadeel Abdelkhaleq, Ayat Taqash, Maysa Al-Hussaini

**Affiliations:** ^1^Department of Pathology, King Hussein Cancer Center, Amman, Jordan; ^2^MedStar Washington Hospital Center/Georgetown University, USA; ^3^University of Jordan School of Medicine, Jordan; ^4^Office of Scientific Affairs and Research, King Hussein Cancer Center (KHCC), Amman, Jordan

## Abstract

**Purpose:**

Microsatellite instability (MSI) caused by mismatch repair protein (MMRP) deficiency is detected in 15% of sporadic colorectal cancers (CRCs). Our aim is to investigate the frequency of MMRP deficiency in young CRC patients, using immunohistochemical analysis.

**Methods:**

This study targeted cases of CRC at King Hussein Cancer Center from 2004 until 2012 in patients 45 years of age or younger at the time of diagnosis. Clinicopathological data was obtained from 155 patients' records. Immunohistochemistry for MLH1, MSH2, PMS2, and MSH6 proteins was performed on paraffin-embedded tissue containing carcinoma.

**Results:**

The median age of patient at diagnosis was 38 years. A total of 29 (19%) cases showed deficient MMRP(dMMRP)expression. Loss of expression of PMS2 was seen in 17 cases, 12 cases of which showed loss of MLH1 expression. Loss of expression of MSH6 was seen in 10 cases, 9 of which showed loss of MSH2 expression. One case (3.4%) showed loss of all four MMR proteins, and another case (3.4%) showed loss of PMS2/MLH1 and MSH6. There was a significant association between abnormal MMR protein expression and tumor location proximal to splenic flexure (*p* value 0.000), pathologic features suggestive of microsatellite instability (*p* value 0.000), P53 negativity (*p* value 0.000), and stage (*p* value 0.02). Patients with dMMRP CRC appeared to have a significantly better overall survival compared to patients with proficient MMRP(pMMRP)(*p* value 0.02). Loss of MSH2/MSH6 was significantly associated with positive family history of cancer (*p* value = 0.020).

**Conclusions:**

The prevalence of dMMRP tumors in this age group appears to be similar to international literature. dMMRP tumors tends to be associated with earlier stages and better outcomes compared to pMMRP cases. dMMRP can serve as a biomarker for better prognosis. These results are of value in directing the clinical management of young patients with CRC.

## 1. Introduction

Colorectal cancer (CRC) is the third most common cancer in men and the second in women worldwide. In the latest worldwide estimates of cancer incidence in 2018, there were over a million cases of colon cancer and about 700.000 cases of rectal cancer in both men and women [1]. However, incidence rates of colorectal carcinoma vary significantly in different areas. According to the Jordan National Cancer Registry (JNCR) report in 2016, there were 641 colorectal cancer cases, accounting for 10.7% of all newly diagnosed cases among Jordanians. It ranked the second among all new cancers in both genders, the first among males and the second among females [2].

With a median age of 68 years in men and 72 in women for colon cancer and 63 years for rectal cancer in both men and women, and as CRC incidence increases significantly beyond the fifth decade of life, screening is usually not recommended for individuals at an average risk younger than 45 years [3, 4]. However, an increase in the incidence of CRC in younger adults has been witnessed recently, with reports from USA suggesting that approximately 11% of colon cancers and 18% of rectal cancers occur in individuals younger than the age of 50 [5–7]. Furthermore, in contrast to the noticed decline in CRC mortality among adults aged 55 years and older, there is about 11% increase in mortality among adults younger than 55 years [4].

Numbers suggest a possible high incidence of CRC in young Jordanian patients, with 102 of CRC diagnosed cases (16%) at the age of 44 years or younger in the most recent registry data in 2014 [2]. At King Hussein Cancer Center, 688 out of 1902 (36.2%) patients diagnosed with CRC were younger than 45 years during the same time period (unpublished data).

Colorectal carcinoma occurs sporadically in the majority of cases. Twenty–30% of all CRCs appear to have a familial basis, and only 5%–10% are due to inherited mutations in well-known cancer-related genes. Recognized hereditary conditions include familial adenomatous polyposis (FAP) syndrome, nonpolyposis hereditary colorectal carcinoma (NPHCC) syndrome, or Lynch syndrome, MUTYH-associated polyposis, and certain hamartomatous polyposis conditions. These syndromes can predispose individuals to the development of CRC at a higher frequency and at a younger age [8].

Hereditary nonpolyposis colorectal cancer (Lynch syndrome)—an autosomal dominant syndrome—is the most common form of hereditary colorectal cancer. It accounts for 2–4% of all colorectal cancers [9], and the lifetime risk for development of CRC is 25–75% [10]. Research showed that individuals from the general population have a 2% lifetime risk of developing CRC, whereas the risk for patients with Lynch syndrome is over 80% [11]. In families with Lynch syndrome, CRC is frequently diagnosed at a younger age and progress more rapidly. About one fourth of family members develop CRC by the age of 50. Adenomatous polyps in affected individuals can progress to invasive cancer within 30 months compared to 10 years in the general population [12]. Extracolonic malignancies associated with Lynch syndrome include endometrial, gastric, ovarian, biliary, urinary tract, small bowel, brain, and pancreatic cancer among others [13]. The most common is endometrial carcinoma, where women affected by the syndrome have up to a 71% lifetime risk of developing endometrial cancer, compared to 1.5% lifetime risk in the general population [11]. Accordingly, detailed criteria have been developed to identify patients with Lynch syndrome including the Amsterdam Criteria, the Bethesda Guidelines, and the revised Bethesda Guidelines [14]. Young age at onset is usually included among the indicators of an inherited CRC syndrome. Therefore, when dealing with an early onset CRC patient, testing for the possibility of Lynch syndrome is warranted.

The hallmark of tumors in Lynch syndrome is microsatellite instability (MSI). Microsatellites are genomic regions in which mutation can occur during DNA replication. These mutations are usually repaired by the mismatch repair proteins(MMRP) [9]. The mechanism of development of CRC in Lynch syndrome is related to germline mutation in one of the DNA mismatch repair (MMR) genes MLH1, PMS2, MSH2, or MSH6 [10]. A cost-effective approach for identifying Lynch syndrome is to perform tumor testing when any of the Bethesda guidelines are identified. The most commonly used method is to begin with MSI and/or immunohistochemistry (IHC) analysis of CRC. Applying immunohistochemical stains on paraffin-embedded tumor tissue sections to look for mutations in one or more of the MMRPs, including MLH1, PMS2, MSH2, and MSH6 proteins, is an easy and convenient method to test for the mutations in tumors associated with loss or inactivation of the relevant mismatch repair gene. The sensitivity of IHC is comparable to that of MSI analysis, with the advantage of direct genetic testing to the appropriate MMR gene when loss of MMRP expression is identified [15]. Additionally, the same method can be used as a screening tool for colorectal cancer patients. The finding of an abnormal MMRP expression in an adenomatous polyp from a patient with a concerning family history could provide justification for formal genetic evaluation [16]. Of note, MSI is detected in about 15% of all CRCs; 3% of these are associated with Lynch syndrome, and the other 12% are caused by sporadic, acquired hypermethylation [17].

In this study, we investigate the prevalence of dMMRP in young patients with CRC and describe patients' and tumors' characteristics and outcome in comparison to pMMRP tumors in the same age group.

## 2. Materials and Methods

This study targeted cases of CRC diagnosed and treated at the King Hussein Cancer Center from 2004 until 2012 in patients 45 years of age or younger at the time of diagnosis. After obtaining the Institutional Review Board approval, all patients' charts were reviewed. The clinical data including the age at diagnosis, the presenting symptoms and signs, family history of colorectal cancer or other types of cancer, the location, stage, and the treatment modality as well as the event-free survival (EFS) and overall survival (OS) were collected.

The pathology slides for the CRC resection specimens were reviewed. Slides containing the tumor and normal tissue and their corresponding paraffin blocks were retrieved when available; otherwise, the biopsy blocks were used instead.

Immunohistochemistry analysis was performed using the following monoclonal antibodies: anti-MLH-1 (clone M1, Roche), anti-MSH2 (clone G219-1129, Roche), anti-MSH6 (clone 44, Roche), anti-PMS2 (clone EPR3947, Roche), and P53 (clone DO7, Roche). Staining was performed using the automated tissue staining system (immunostainer BenchMark ULTRA (Ventana Medical systems, Inc.)) using validated protocols. Testing for PMS2 and MSH6 was done first [18]. Only cases with aberrant expression of PMS2 and/or MSH6 were tested for MLH1 and/or MSH2, respectively. Aberrant expression was considered when the tumor showed loss of nuclear staining for any of the MMRP, in the presence of an internal positive control (endothelium or lymphocytes). Two pathologists (B. M and M. H) assessed all cases independently. The few cases with discrepant scoring were reevaluated jointly, and agreement was reached in all cases.

Structured spreadsheets for data collection and Access Database were developed to assist reviewing and documenting epidemiological and clinical factors including follow-up and survival data. Descriptive statistics using frequencies and percentages were applied. Univariate analysis was performed using the Student *t*-test for continuous variables, and the differences in proportions were tested with the *χ*^2^ (Chi-square) or Fisher exact test. Multivariate correlation analysis was performed using the logistic regression test.

The Kaplan-Meier method was adopted to estimate EFS and OS curves, and the log-rank test was used to compare patients' survival times. The OS was calculated from the primary diagnosis to death from any cause; patients who were alive at the last follow-up were censored at that time. The EFS was calculated from the primary diagnosis to the first event (relapse or death). Survival was expressed as median with a 95% confidence interval. A significance criterion of *p* ≤ 0.05was used in the analysis. All analyses were performed using SAS version 9.4 (SAS Institute Inc., Cary, NC).

## 3. Results

A total of 155 cases with available paraffin blocks were included in the study. The median age at diagnosis was 38 years (range from 17 to 45 years). Eighty three (54%) patients were male, and male to female ratio was 1.15 : 1. Most tumors included in the study were located in the rectum (47%), with 15%, 11%, and 25% located in the right, left, and sigmoid colon, respectively. Most tumors were advanced at the time of diagnosis, with approximately 70% presenting at stage III and IV disease. The main histopathological features of the tumors were as follows: 132 patients (85.2%) had well-moderately differentiated adenocarcinoma, 23 (14.8%) patients had poorly differentiated adenocarcinoma, and 27 (17.4%) patients had mucinous carcinoma. The clinicopathological features are summarized in Table 1.

**Figure 1:**
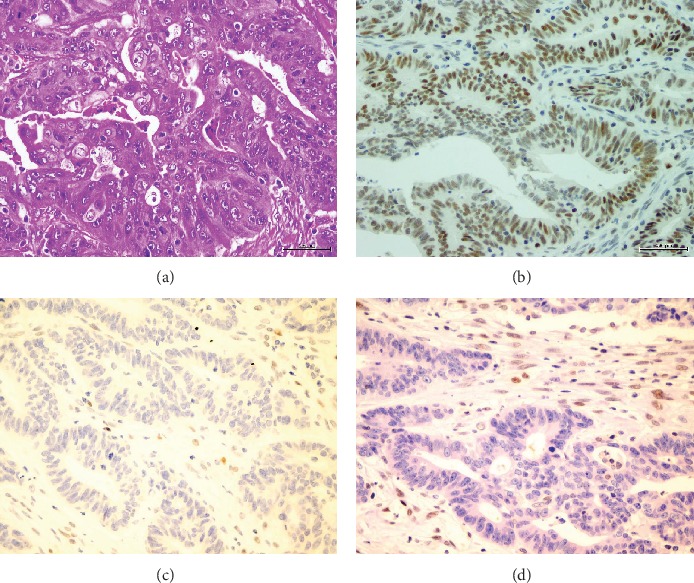
Immunohistochemical staining for mismatch repair proteins. (a) Moderately differentiated adenocarcinoma (H&E ×400). (b) PMS-2 immunostain shows retained nuclear staining in the tumor (×400). (c) MSH-6 immunostain shows loss of nuclear staining in the tumor, while it is retained in infiltrating lymphocytes (×400). (d) MSH2 also shows loss of nuclear staining in the tumor (×400).

**Figure 2:**
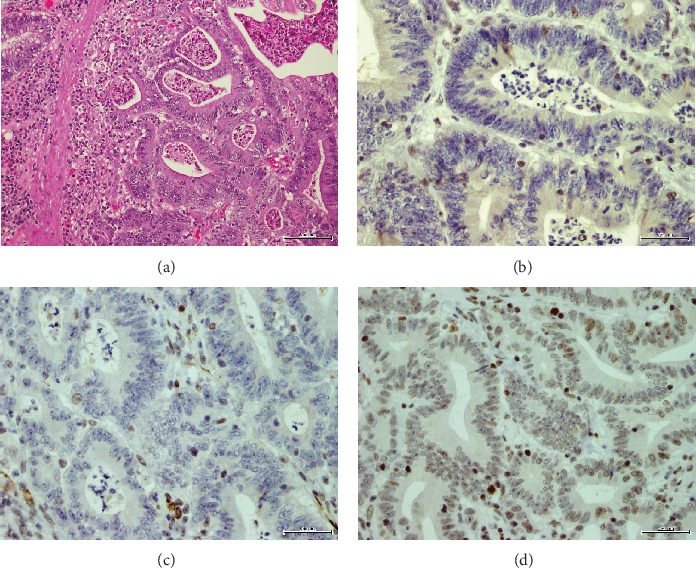
Immunohistochemical staining for mismatch repair proteins. (a) Moderately differentiated adenocarcinoma (H&E ×400). (b) PMS-2 immunostain shows loss of nuclear staining in the tumor, while it is retained in infiltrating lymphocytes (×400). (c) MLH-1 immunostain shows loss of nuclear staining in the tumor, while it is retained in infiltrating lymphocytes (×400). (d) MSH-6 immunostain is retained in tumor cell nuclei (×400).

A total of 29 (19%) cases were dMMRP. Loss of expression of PMS2 was seen in 17 cases, 12 of which showed loss of MLH1 expression. Additionally, loss of expression of MSH6 was seen in 10 cases, 9 of which showed loss of MSH2 expression. One case showed loss of all four MMR proteins, and another case showed loss of PMS2/MLH1 and MSH6.  Table 2 summarizes the characteristics of individual patients with mismatch repair deficiency. Figures 1 and 2 show examples of the MLH1/PMS2 and MSH2/MSH6 loss of nuclear staining, respectively.

There was a significant association between dMMRP and tumor location proximal to splenic flexure, as well as the pathologic features suggestive of MSI (mucinous component, tumor-infiltrating lymphocytes (TIL), and Crohn's-like lymphocytic reaction (CLR)). MMRP-deficient tumors presented significantly at a lower stage and with negative nodal metastasis. About 86% of dMMRP cases were negative for P53 which was statistically significant (*p* value = 0.000).  Table 3 presents the relationship between the dMMRP tumors and the various clinicopathological features. Interestingly, loss of MSH2/MSH6, but not MLH1/PMS2, was significantly associated with positive family history in first- and/or second-degree relatives (*p* value = 0.020). On multivariate analysis, right-sided location, pathologic features suggestive of MSI, P53 negativity, early stage, and survival status remained significantly associated with dMMRP (Table 4).

**Figure 3:**
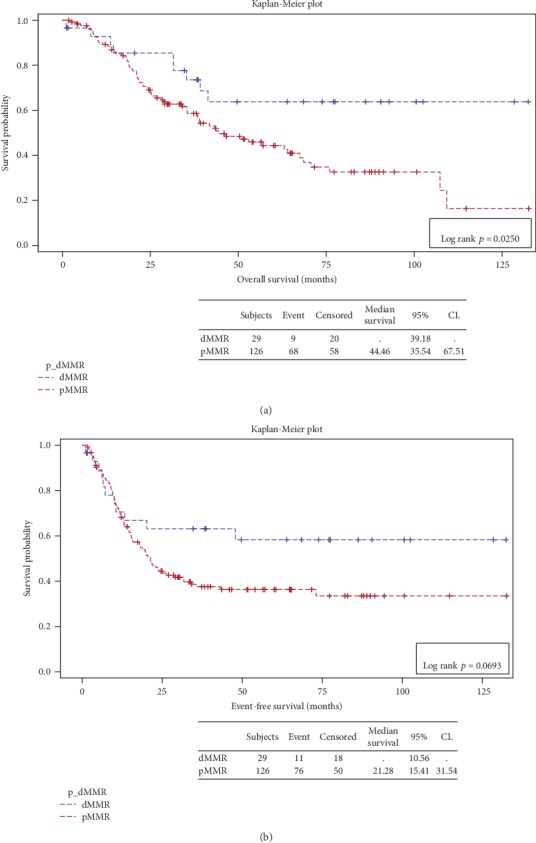
Survival curves for overall survival (a) and event-free survival (b) in patients with dMMRP vs. patients with pMMRP colorectal carcinoma.

The Kaplan-Meier survival curves of patients with dMMR or pMMR indicated a significantly better OS but not EFS in patients with dMMR (*p* value 0.0250 and 0.0693, respectively) (Figure 3).

## 4. Discussion

In Jordan, colorectal cancer replaced lung cancer as the most common cancer in men and the second most common cancer in women. The rates of colorectal cancer increased significantly in Jordan which may be attributed to changes in diet habits and the westernization of the lifestyle [19, 20]. Additionally, Tamwneh et al. reported a low rate of colorectal cancer under 40 years of age in Jordan between 1996 and 2005, after which the rates began to rise steadily, with rates of 18.7 and 24.3 per 100 000 poplutation for males and females, respectively, by the age of 40–59 years [20]. After this period reports from Jordan cancer registry showed increased trends of age-specific incidence rates (ASIR) and age-standardized incidence rates (ASR) for colorectal cancer [2].

Rectal cancer is far more common than colonic cancer. According to the Jordan National Cancer Registry during the period of the study(2004-2012), there were a total of 4453 colorectal cancer cases, 35.0% of which were in the rectum [2]. Additionally, rectal carcinoma accounted for 37.8% (*n* = 719) out of 1902 CRC patients over the same time period at KHCC (unpublished data). We included 155 cases in this study of which around 47% were located in the rectum. This finding may suggest that CRC in young individuals have a predilection for the distal colon, regardless of MMRP status. A Similar finding was previously reported by Goel et al. [21].

Studies of colorectal carcinogenesis suggest that molecular events which lead to colonic adenocarcinoma are heterogeneous and include genetic and epigenetic abnormalities. The commonest genetic pathways described are the APC/*β*-catenin pathway and the MSI pathway, which are associated with defects in DNA mismatch repair and accumulation of mutations in microsatellite repeat regions of the genome. Epigenetic events via methylation-induced gene silencing may enhance progression along either pathways [22].

Several studies addressed the frequency of MMRP deficiency either by IHC and molecular studies or by IHC alone. Such studies reported a highly variable prevalence of MMRP deficiency, probably attributed to differences in the cut-off age and methods of screening used. When taking the age into account, the rate of MMRP deficiency reported in this study (19%) is comparable with that of other studies that examined the prevalence of dMMRP among young patients (<50 years old) [21, 23–25]. In the Middle East region, however, Ashktorab et al. reported a rate of 16.3% dMMRP by IHC in Omani subjects [26]. Using MSI as the initial screening test, a study from Saudi Arabia reported 11.6% MSI cases [27]. In the Iranian population, 10.57% of the early-onset CRCs were dMMRP [28]. A low frequency (8.4%) of dMMRP was reported in a study from Japan [29], which was the first to evaluate the prevalence of dMMRP in CRC in the young Japanese population, suggesting that this low rate might be related to yet-to-be identified genetic or environmental factors.

Histologic features of MSI-H are defined as the presence of tumor-infiltrating lymphocytes, Crohn's-like lymphocytic reaction, and mucinous differentiation [30] and are well described in the literature. Analysis of the relationship between dMMRP and these features showed significant association in our study. Sporadic MSI-H CRCs are also known to be characterized by eosinophilic tumor cells (ETC) [31]; however, our study failed to show a significant association.

As this study sample included early-onset CRC cases, it should be expected to include sporadic as well as familial cases. Sporadic dMMRP cases are often associated with hypermethylation of the promoter region of the MLH1 with resultant silencing of MLH1 gene and absent protein expression by IHC [22]. In keeping with this idea and since sporadic dMMRP CRC cases constitute a major proportion of patients, our study showed loss of MLH1 as the most common pattern of dMMRP tumors accounting for 41% of cases. Conversely, loss of expression of MSH2, MSH6, or PMS2 in isolation is considered a strong evidence of a germline mutation in the respective gene [32]. Of these, MSH2 is the most commonly mutated in Lynch syndrome accounting for 41% of cases [33]. The current study showed loss of MSH2 in 30%, isolated MSH6 in 3%, and isolated PMS2 in 17% of dMMRP cases. Notably, there were no cases of constitutional mismatch deficiency in our study. This is defined by loss of staining of MMRP in tumor cells as well as normal tissue. Individuals with this pattern have biallelic germline mutations in MMR genes [34]. Moreover, only loss of MSH2/MSH6 showed a significant association with positive family history (*p* value = 0.020) supporting the idea of germline mutation.

Univariate analysis showed a significant association between tumor location proximal to splenic flexure (i.e., right-sided location) and MMRP deficiency. This association remains significant also on the multivariate analysis with an odds ratio of 5.462. This finding is well documented and aligned with the literature [21, 25, 29, 35–38].

We found that dMMRP tumors tended to present at an earlier stage in consistency with several previous studies of MMRP-deficient colorectal cancer [25, 35, 37, 39]. In this study, there was no significant association between the stage of the primary tumor (T stage) or distant metastasis (M stage) and MMRP deficiency. However, a significant association was seen in nodal status, with dMMRP cases tending to have less nodal metastasis than pMMRP cases (*p* value = 0.035). This low frequency of lymph node involvement is the characteristics of dMMRP [38]. Multivariate analysis confirmed that dMMRP cases have a lower rate of stage III disease when compared to stages I and II with odds ratio (4.587; 95% CI (confidence interval), 1.190-17.685; *p* = 0.0269).

In multivariate analysis, another variant that remained significant is the P53 status, with the tendency of dMMRP cases to be P53 negative [40, 41].

At the last available follow-up, 19 out of the 29 dMMR cases (66%) were alive without disease, 1 (3%) was alive with metastasis, and 9 (31%) were dead of disease. The Kaplan-Meier survival curves showed a significantly better OS in dMMRP compared to pMMRP patients, consistent with previously described outcome [37, 42, 43].

We acknowledge limitations in this study including the retrospective nature of the data, with all patients collected from a single cancer center, which might induce referral bias. Also, we did not perform germline mutational analysis to confirm patients with Lynch syndrome. However, this study sheds light on the frequency of dMMRP in young patients with CRC in our population. Our findings including the suggestive microscopic features,the better outcome and the lower stage are consistent with the international literature. In a country with limited resources, but with a prevalence of CRC in the young as frequent as in western countries, testing for MMRP by a cost-effective method like IHC is recommended. Further confirmatory testing, genetic counselling, and surveillance for the patient and family members can be guided by these results.

## Figures and Tables

**Table 1:** Clinicopathological criteria of patients with colorectal cancer.

Clinicopathological features	*N*	%
Gender		
Male	83	53.5%
Female	72	46.5%
Age (years) classification		
20 or less	1	0.6%
21-25	4	2.6%
26-30	12	7.7%
31-35	37	23.9%
36-40	56	36.1%
41-45	45	29.0%
Median age	38	
Location		
Right colon	23	14.8%
Transverse colon	3	1.9%
Left colon	17	11.0%
Sigmoid	39	25.2%
Rectum	73	47.1%
Histologic features		
Well-differentiated adenocarcinoma	4	2.6%
Moderately differentiated adenocarcinoma	128	82.6%
Poorly differentiated adenocarcinoma	23	14.8%
Mucinous component	27	17.4%
Lymphovascular invasion	55	35.5%
Eosinophilic tumor cells	10	6.5%
TNM stage groupings		
I	12	7.7%
II	35	22.6%
III	67	43.2%
IV	41	26.5%

**Table 2:** Characteristics of individual patients with mismatch repair deficiency.

	Age (Yr)	Gender	Family history	Tumor location	Stage	Grade	Pathological feature suggestive of MSI	Specific pathologic pattern	Pattern of MMR loss	Outcome
1	43	M	NA	Ascending	I	II	Yes	TIL, CLR	MLH1/PMS2	AWD
2	44	M	No	Sigmoid	III	II	Yes	CLR	MLH1/PMS2	AWD
3	42	F	Yes (1^st^ degree)	Sigmoid	III	II	Yes	TIL, CLR	MLH1/PMS2	AWD
4	42	F	No	Rectum	III	II	Yes	CLR	MLH1/PMS2	AWD
5	44	M	Yes (1^st^ degree)	Rectum	I	II	No	—	MSH2/MSH6	AWD
6	42	M	No	Ascending	III	II	No	—	MLH1/PMS2	AWD
7	34	M	Yes (2^nd^ degree)	Sigmoid	II	II	Yes	TIL, CLR	MSH2/MSH6	AWD
8	35	M	Yes (1^st^ degree)	Rectum	I	II	Yes	CLR	MSH2/MSH6	AWD
9	33	M	NA	Ascending	II	II	Yes	Mucinous, TIL, CLR	PMS2	AWD
10	39	M	NA	Ascending	II	II	Yes	Mucinous	MSH2/MSH6	AWD
11	34	M	NA	Ascending	IV	II	Yes	Mucinous, TIL, CLR, ETC	MLH1/PMS2	AWMD
12	38	F	No	Rectum	IV	III	Yes	Mucinous	PMS2	DOD
13	30	M	Yes (1^st^ degree)	Transverse	III	III	Yes	TIL, CLR, ETC	MSH2/MSH6	DOD
14	40	M	NA	Rectum	II	II	Yes	Mucinous	MLH1/PMS2	DOD
15	30	F	NA	Descending	IV	II	No	—	PMS2	DOD
16	36	F	No	Sigmoid	II	II	No	—	PMS2	AWD
17	35	M	Yes (2^nd^ degree)	Rectum	IV	II	No	—	MLH1/PMS2	DOD
18	37	F	No	Ascending	II	II	Yes	CLR	MLH1/PMS2	DOD
19	40	M	Yes (1^st^ degree)	Ascending	IV	II	Yes	Mucinous	MSH2/MSH6	DOD
20	33	M	Yes (2^nd^ degree)	Ascending	II	II	No	—	All 4	AWD
21	34	F	Yes (1^st^ degree)	Ascending	II	II	No	—	MSH2/MSH6	AWD
22	37	M	No	Sigmoid	II	II	Yes	TIL	MSH6	AWD
23	38	F	Yes (1^st^ degree)	Ascending	III	II	Yes	Mucinous	MLH1/PMS2	AWD
24	37	M	No	Descending	IV	III	Yes	Mucinous	MLH1/PMS2	DOD
25	26	F	No	Descending	II	I	Yes	CLR	MSH2/MSH6	AWD
26	37	M	No	Descending	III	III	Yes	Mucinous	PMS2	DOD
27	36	F	NA	Ascending	II	II	Yes	CLR	MLH1/PMS2	AWD
28	37	M	NA	Descending	II	II	Yes	CLR	MLH1/PMS2 and MSH6	AWD
29	37	M	NA	Rectum	III	I	Yes	Mucinous	MSH2/MSH6	AWD

Abbreviations: Yr: year; M: male; F: female; NA: not available; TIL: tumor infiltrating lymphocytes; CLR: Crohn's-like reaction; AWD: alive without disease; DOD: dead of disease; AWMD: alive with metastatic disease.

**Table 3:** Relationship between the status of MMRP expression and the clinicopathological features of colon cancer.

Clinicopathological features	Total cases (*n*)	dMMRP *n* (%)	pMMRP *n* (%)	*p* value
Gender				
Male	83	19 (65.5%)	64 (50.8%)	0.152
Female	72	10 (34.5%)	62 (49.2%)	
Age				
17-38	83	20 (69.0%)	63 (50.0%)	0.065
39-45	72	9 (31.0%)	63 (50.0%)	
Neo/adj_treatment				
Yes	31	1 (3.4%)	30 (23.8%)	0.010
No	124	28 (96.6%)	96 (76.2%)	
Location				
Proximal to splenic flexure	26	12 (41.4%)	14 (11.1%)	0.000
Distal to splenic flexure	129	17 (58.6%)	112 (88.9%)	
Degree of differentiation				
Well-moderately differentiated (I and II)	132	25 (86.2%)	107 (84.9%)	1.000
Poorly undifferentiated adenocarcinoma (III and IV)	23	4 (13.8%)	19 (15.1%)	
Pathologic features suggestive of MSI				
Present	62	22 (75.9%)	40 (31.7%)	0.000
Absent	93	7 (24.1%)	86 (68.3%)	
Mucinous component				
Present	27	10 (34.5%)	17 (13.5%)	0.007
Absent	128	19 (65.5%)	109 (86.5%)	
Tumor infiltrating lymphocytes (TIL)				
Present	18	7 (24.1%)	11 (8.7%)	0.020
Absent	137	22 (75.9%)	115 (91.3%)	
Crohn's-like reaction (CLR)				
Present	29	14 (50.0%)	15 (14.0%)	0.000
Absent	106	14 (50.0%)	92 (86.0%)	
Cannot be determined^∗^	20			
Eosinophilic tumor cells (ETC)				
Present	10	2 (6.9%)	8 (6.3%)	1.000
Absent	145	27 (93.1%)	118 (93.7%)	
P53 status				
Positive	85	4 (13.8%)	81 (64.8%)	0.000
Negative	69	25 (86.2%)	44 (35.2%)	
Vascular invasion				
Present	55	8 (28.6%)	47 (42.0%)	0.194
Absent	85	20 (71.4%)	65 (58.0%)	
Cannot be determined^∗^	15			
T stage				
Tx	10	1 (3.4%)	9 (7.1%)	0.784
T1/T2	19	4 (13.8%)	15 (11.9%)	
T3/T4	126	24 (82.8%)	102 (81.0%)	
Node status				
Nx	11	1 (3.4%)	10 (7.9%)	0.035
N0	49	15 (51.7%)	34 (27.0%)	
N1/N2	95	13 (44.8%)	82 (65.1%)	
M stage				
M0	114	23 (79.3%)	91 (72.2%)	0.435
M1	41	6 (20.7%)	35 (27.8%)	
TNM stage groupings				
I and II	47	15 (51.7%)	32 (25.4%)	0.020
III	67	8 (27.6%)	59 (46.8%)	
IV	41	6 (20.7%)	35 (27.8%)	
Outcome				
Alive	78	20 (69.0%)	58 (46.0%)	0.026
Dead	77	9 (31.0%)	68 (54.0%)	
Family history of cancer				
Present (1^st^ and/or 2^nd^ degree)	52	10 (50.0%)	42 (42.4%)	0.533
Absent	67	10 (50.0%)	57 (57.6%)	
Cannot be determined^∗∗^	36			

^∗^Cases were evaluated on biopsy specimen in which these particular characteristics could not be determined. ^∗∗^Family history was not available for these cases.

**Table 4:** Multivariate analysis of relationship between deficient mismatch repair and clinico-pathological features of colon cancer.

Effect	Odds ratio	95% Wald confidence limits		*p* value
Neo-adjuvant treatment (no vs. yes)	5.429	0.530	55.576	0.1540
Location (right vs. left sided)	5.462	1.469	20.313	0.0113
Pathologic features (yes vs. no)	12.048	3.413	41.667	0.0001
P53 status (negative vs. positive)	18.591	3.830	90.251	0.0003
Stage (III vs. “I and II”)	4.587	1.190	17.685	0.0269
Stage (IV vs. “I and II”)	0.348	0.053	2.293	0.2727
Status (alive vs. dead)	9.052	1.855	44.173	0.0065

## Data Availability

The data used to support the findings of this study are available from the corresponding author upon request.
